# Ganglion cell-inner plexiform layer thickness by swept-source optical coherence tomography in healthy Korean children: Normative data and biometric correlations

**DOI:** 10.1038/s41598-018-28870-4

**Published:** 2018-07-13

**Authors:** Yoon Pyo Lee, Young-Su Ju, Dong Gyu Choi

**Affiliations:** 1 Department of Ophthalmology, Kangnam Sacred Heart Hospital, Hallym University College of Medicine, Seoul, Korea; 2 Department of Occupational and Environmental Medicine, Hallym University Sacred Heart Hospital, Anyang, Korea

## Abstract

The purpose of this study was to identify the normative values of ganglion cell-inner plexiform layer (GCIPL) thickness in healthy Korean children using swept-source optical coherence tomography (SS-OCT) and to investigate the correlations of age, refractive error, axial length (AL), retinal nerve fiber layer (RNFL) thickness and cup-to-disc (C/D) ratio with GCIPL thickness. Children aged between 3 and 17 who had visited our pediatric ophthalmology clinic were enrolled. Each subject underwent full ophthalmic examinations including RNFL thickness, C/D ratio and GCIPL thickness measurement by SS-OCT as well as AL measurement by partial-coherence interferometry. A total of 254 eyes of 127 children were included. The mean average GCIPL thickness was 71.5 ± 5.35 *μ*m; the thickest sector was the superonasal and the thinnest the inferior. According to multivariate regression analysis, average GCIPL thickness was significantly associated with spherical equivalent and RNFL thickness (P < 0.0001 for both): the higher the myopia or the thinner the RNFL thickness, the thinner the GCIPL thickness. In conclusion, this study provides an SS-OCT-based pediatric normative database of GCIPL thickness that can serve as a reference for early detection and follow-up of glaucoma and optic nerve diseases in children.

## Introduction

Optical coherence tomography (OCT) is a noninvasive, noncontact and objective high-resolution cross-sectional tissue imaging technique^1^ that has been widely used in recent years in the diagnosis and follow-up of glaucoma, macular diseases and optic nerve diseases. Traditional OCT was a time-domain OCT (TD-OCT)^2,3^ by which depth information on the retina was obtained after the longitudinal translation of a reference arm. Spectral-domain OCT (SD-OCT), introduced in the 2000s, measures the interferometric signal detected as a function of optical frequency, thereby allowing for imaging speeds 50 times faster than TD-OCT and providing a greater number of images per unit area^4–6^. Swept-source OCT (SS-OCT), meanwhile, is a new technique employing a fast-wavelength-scanning light source. SS-OCT, compared with the current SD-OCT, offers greater sensitivity at greater scanning depths^7^. OCT’s relative ease, speed and objective-value provision, moreover, make it particularly advantageous over visual-field testing, which, requiring patients’ cooperation for acquisition of reliable and reproducible examination results, is difficult with young children.

Lately, the development of OCT has enabled the measurement of the retinal ganglion cell layer as well as the peripapillary retinal nerve fiber layer (RNFL). Recent studies have shown that the assessment of the macular ganglion cell inner plexiform layer (GCIPL) can be an especially effective means of evaluating glaucomatous damage^8,9^. Further, some studies have suggested that GCIPL is superior to RNFL thickness measurement for detection of early glaucoma^10–12^. Besides, measurement of macular GCIPL thickness has been shown to be useful for estimation of early axonal loss in optic neuropathies such as optic neuritis or nonarteritic anterior ischemic optic neuropathy^13,14^. Such measurement also can be helpful for detection of early retinal toxicity due to hydroxychloroquine or early optic nerve change due to ethambutol^15,16^.

Likewise, in situations where the importance of macular GCIPL thickness has been emphasized, its normative data should be obtained in order to evaluate optic nerve, macular diseases or glaucomatous damage. Although many studies have reported normative data on macular GCIPL thickness in adults^17–19^, a few have provided such data on children. Therefore, we undertook the present study to identify, by SS-OCT, normative data on the macular GCIPL in healthy Korean children and also to investigate their correlations with biometric factors such as age, refractive error (spherical equivalent, SE), axial length (AL), RNFL thickness and cup-to-disc (C/D) ratio.

## Methods

### Study design and subjects

We retrospectively reviewed the medical records of 127 Korean children (254 eyes) aged between 3 and 17 who had visited our pediatric ophthalmology clinic between December 2015 and March 2017 and had no ocular abnormalities except refractive errors. We routinely performed comprehensive ocular examinations including OCT measurements, if possible, in children visiting the clinic for the first time. The exclusion criteria in this study were as follows: any evidence of macular or optic nerve abnormality, glaucoma or intraocular pressure (IOP) ≥ 22 mmHg, best-corrected visual acuity worse than 20/40, history of prematurity or systemic diseases, history of intraocular or periocular surgery, and patient or parental refusal to undergo, or poor cooperation in undergoing, ocular examinations including GCIPL measurements. Each subject underwent initial ophthalmic examinations including measurement of best-corrected visual acuity, IOP, cycloplegic refraction, assessment of ocular motility and alignment, and measurement of GCIPL, AL, RNFL thickness and C/D ratio. The pupils were dilated with cyclopentolate 1% and tropicamide 1% at intervals of 15 minutes, and manual cycloplegic refraction as well as autorefraction by autokeratorefractometry (KR-800A^®^, Topcon, Japan) was assessed 60 min after instillation of the first drop. Axial length was measured using partial-coherence interferometry (Lenstar LS-900^®^, Haag-Streit AG, Switzerland) before cycloplegia, and the mean of 5 measurements was used in the subsequent analysis. This study’s protocol adhered to the Declaration of Helsinki and was approved by the Institutional Review Board of Hallym University Medical Center (2017-11-007). After explanation of the nature and possible consequences of the study, written informed consent was obtained from each subject’s parent or legal guardian before inclusion.

### Optical coherence tomography scanning procedure

**Figure 1:**
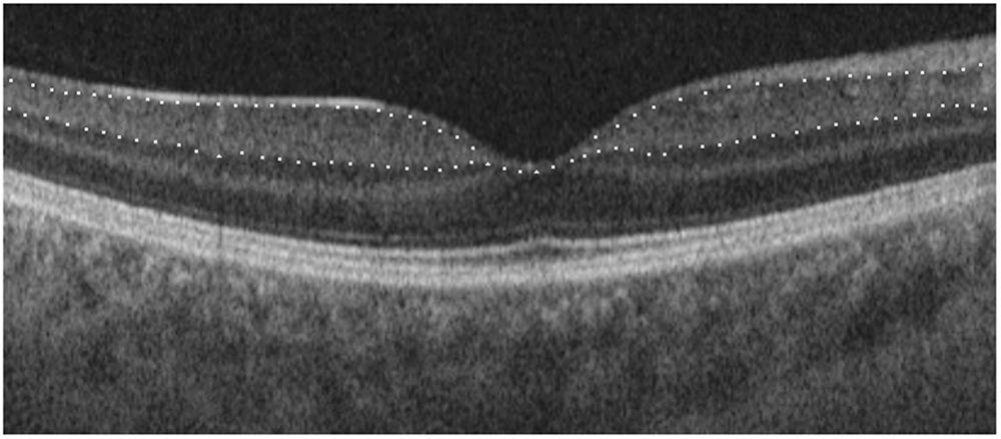
Macular SS-OCT with intraretinal layer segmentation from horizontal scan. Upper white dotted line: junction of RNFL and ganglion cell layer; lower white dotted line: junction of inner plexiform layer and inner nuclear layer.

**Figure 2:**
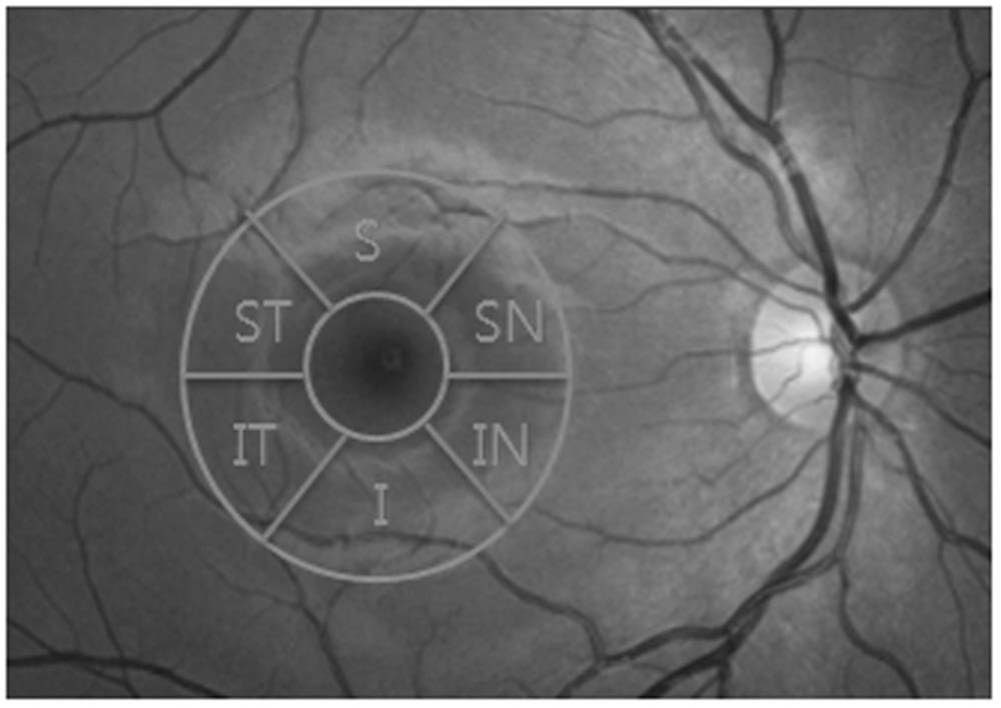
Projection image of macula-centered SS-OCT volume with annular sectors, used for computing of regional GCIPL.

All of the imaging procedures were performed using SS-OCT (DRI OCT Triton^®^, Topcon, Japan). Two scans, including 1 macular scan centered on the fovea (3D-Macula 7 × 7 mm protocol) and 1 peripapillary scan centered on the optic disc (3D-Disc 6 × 6 mm protocol), were acquired from both eyes of the same subject. The GCIPL between the outer boundary of the RNFL and the outer boundary of the inner plexiform layer was measured at the macular region on SS-OCT images using a specific automatic segmentation algorithm (Fig. 1). The average and 6 sectorial (superior, superonasal, inferonasal, inferior, inferotemporal, superotemporal) GCIPL thicknesses were measured from the circular annulus centered on the fovea (Fig. 2). On the basis of the obtained OCT images, the following parameters also were measured automatically: peripapillary RNFL thickness, area C/D ratio, horizontal C/D ratio, and vertical C/D ratio.

All of the OCT scans were taken by three trained technicians prior to pupil dilation. Only high-quality scans, which is to say those with a TopQ Image Quality ≥ 60 and without involuntary saccade or blinking artifacts, were used for the analysis.

### Main outcome measures

The main outcome measures were average and sectorial GCIPL thicknesses of healthy children by SS-OCT as normative data. Correlations between GCIPL thickness and several biometric factors including age, SE, AL, RNFL thickness and C/D ratio were also investigated.

### Statistical analyses

Statistical analyses were performed using SPSS software version 23.0 (IBM-SPSS, Chicago, Illinois, USA). Descriptive statistics were reported as mean ± standard deviation (SD).

The effects of age, SE, AL, RNFL thickness and C/D ratio on average GCIPL thickness were evaluated by univariate and multivariate regression analyses with mixed models, taking correlation between fellow eyes (random intercepts at the subject level) into account^20^. P < 0.05 was considered to represent statistical significance.

The intra-observer repeatability of our measurements was assessed by 37 three-consecutive right-eye measurements of average GCIPL thickness randomly selected from among the OCT scans, and was evaluated by intra-class correlation coefficient (ICC) using a 2-way mixed-effect model of SPSS.

The ICC indicates the proportion of total variability in measurements between different subjects. As such, the ICC is an index of measurement reliability that ranges from 0 to 1, values of 0.81–1.00 indicating almost-perfect agreement^21^.

## Results

A total of 254 eyes of 127 children (62 males and 65 females) were included in this study. The mean age of the subjects was 9.52 ± 3.79 years (range: 3–17 years). The mean SE was −1.19 ± 3.08 D (range: −12.00 – + 7.25 D). The mean AL was 23.3 ± 0.903 mm (range: 20.94–27.49 mm). The mean average RNFL thickness was 108 ± 12.5 *μ*m (range: 58–149 *μ*m). The mean area C/D ratio was 0.326 ± 0.168 (range: 0.01–0.77), horizontal C/D ratio 0.546 ± 0.161 (range: 0.12–0.87), and vertical C/D ratio 0.521 ± 0.156 (range: 0.08–0.89). Table 1 summarizes demographic and biometric characteristics of the subjects.

**Table 1:** Demographic and Ocular Characteristics of Subjects.

		Mean ± SD	Range
Age (y)		9.52 ± 3.79	3–18
SE (D)		−1.19 ± 3.08	−12.00–+7.25
AL (mm)		23.3 ± 0.903	20.94–27.49
RNFL thickness (*μ*m)		108 ± 12.5	58–149
C/D ratio	Area	0.326 ± 0.168	0.01–0.77
	Horizontal	0.546 ± 0.161	0.12–0.87
	Vertical	0.521 ± 0.156	0.08–0.89

SD = Standard deviation, SE = Spherical equivalent, AL = Axial length, RNFL = Retinal nerve fiber layer, C/D ratio = Cup-to-disc ratio.

The mean thickness of the average GCIPL was 71.6 ± 5.36 *μ*m. The superonasal sector (75.1 ± 5.72 *μ*m) was the thickest and the inferior (66.8 ± 5.95 *μ*m) the thinnest (Table 2).

**Table 2:** GCIPL thickness measured by SS-OCT.

	Mean ± SD	Range
Average (*μ*m)	71.6 ± 5.35	49.3–87.2
Superior (*μ*m)	70.6 ± 5.50	44–84
Superonasal (*μ*m)	75.1 ± 5.72	50–90
Superotemporal (*μ*m)	71.8 ± 5.47	53–92
Inferior (*μ*m)	66.8 ± 5.95	35–82
Inferonasal (*μ*m)	72.8 ± 5.90	47–88
Inferotemporal (*μ*m)	72.3 ± 6.08	40–93

GCIPL = Ganglion cell-inner plexiform layer, SS-OCT = Swept-source optical coherence tomography, SD = Standard deviation.

The ICC value of intra-observer repeatability was 0.972 (95% confidence interval, 0.953–0.985, P < 0.001), which was within the “almost-perfect agreement” range.

The results of the univariate and multivariate analyses of the correlations between average GCIPL thickness and the other biometric factors are presented in Table 3. The univariate regression analysis on the mixed model showed that average GCIPL thickness was significantly associated with SE (β, 0.710; P < 0.0001), AL (β, −1.907; P < 0.0001) and average RNFL thickness (β, 0.202; P < 0.0001). In the multivariate regression analysis on the mixed model, average GCIPL thickness was significantly associated with SE (β, 0.491; P < 0.0001) and average RNFL thickness (β, 0.151; P < 0.0001) but not with AL (β, −0.527; P = 0.149): the higher the myopia or the thinner the RNFL thickness, the thinner the GCIPL thickness. For a 1 D increase in SE, the GCIPL thickness increases by 0.491 *μ*m, and for a 1 *μ*m increase in RNFL thickness, the GCIPL thickness increases by 0.151 *μ*m. In both analyses univariate and multivariate, average GCIPL thickness was not affected by age (β, −0.145; P = 0.245 and β, 0.106; P = 0.296, respectively).

**Table 3:** Correlations with average GCIPL thickness and other biometric factors.

		Average GCIPL			
		Univariate regression analysis		Multivariate regression analysis	
		β (95% CI)	P	β (95% CI)	P
Age		−0.145 (−0.319, 0.030)	0.245	0.106 (−0.037, 0.249)	0.296
SE		0.710 (0.508, 0.913)	<0.0001	0.491 (0.291, 0.691)	<0.0001
AL		−1.907 (−2.606, −1.208)	<0.0001	−0.527 (−1.149, 0.095)	0.149
RNFL thickness		0.202 (0.162, 0.242)	<0.0001	0.151 (0.111, 0.191)	<0.0001
C/D ratio	Area	−0.122 (−4.066, 3.822)	0.935		
	Horizontal	−0.359 (−4.474, 3.756)	0.804		
	Vertical	−0.0004 (−4.243, 4.243)	0.999		

GCIPL = Ganglion cell-inner plexiform layer, CI = Confidence interval, SE = Spherical equivalent, AL = Axial length, RNFL = Retinal nerve fiber layer, C/D ratio = Cup-to-disc ratio.

Regression analysis with mixed models: P < 0.05 indicates a statistically significant difference.

## Discussion

Recently, several studies on GCIPL thickness of normal healthy eyes as measured by SD-OCT have been reported^22,23^. Our present results in children with SS-OCT, the latest commercially available version of OCT, showed ICC value of intra-observer repeatability of 0.972 for the average GCIPL thickness. It was comparable not only with the results obtained with previous version of OCT in children but also with those obtained in adults^24–28^.

Totan *et al*.^22^ evaluated healthy Turkish children aged 3 to 17 years using SD-OCT, and found that the average GCIPL thickness was 83.36 ± 5.40 *μ*m, superior GCIPL (the thickest sector) 84.66 *μ*m, and inferior GCIPL (the thinnest sector) 81.95 *μ*m. Goh *et al*.^23^ examined 139 children aged 3 to 18 years using SD-OCT and reported that the average GCIPL thickness was 82.59 ± 6.29 *μ*m, superior 83.68 *μ*m, and inferior 81.64 *μ*m. To the best of our knowledge, there has been no previous report of children’s normative values of GCIPL thickness obtained with SS-OCT. In our study, average GCIPL thickness was 71.6 ± 5.35 *μ*m, superonasal GCIPL (the thickest sector) 75.1 ± 5.72 *μ*m, and inferior GCIPL (the thinnest sector) 66.8 ± 5.95 *μ*m. These values were significantly thinner than in those previous studies noted above, which discrepancy was due to the different OCT versions employed: SS-OCT versus SD-OCT. Yang Z *et al*.^29^ evaluated the diagnostic abilities of SS-OCT- and SD-OCT-obtained macular GCIPL measurements in glaucoma patients and healthy adults aged 38 to 83, and found that the average GCIPL thickness in healthy eyes was 70.5 ± 5.5 *μ*m using SS-OCT and 82.1 ± 6.6 *μ*m using SD-OCT. They concluded that those two imaging modalities’ readings are not interchangeable, and posited the reason for this discrepancy as follows: SS-OCT uses a wide-angle scan focusing on the region between the optic disc and macula to measure macular GCIPL and peripapillary RNFL thicknesses, whereas SD-OCT uses separate macular and disc cube scans. Since the nerve fiber layer reflectance can be highly affected by the angle of illumination, the difference in the focus areas during image acquisition between SS-OCT’s wide-angle scan and SD-OCT’s disc or macular scan might have an impact on their GCIPL and RNFL readings. Besides the differences in the two OCT scan methods, other factors such as scan quality might also contribute to small discrepancies in GCIPL and RNFL measurements^29^.

In the present study, we additionally investigated the correlations between average GCIPL thickness and age, SE, AL, RNFL thickness, and C/D ratio. In the multivariate regression analysis, we found that average GCIPL thickness was significantly associated with SE (β, 0.491; P < 0.0001) and average RNFL thickness (β, 0.151; P < 0.0001), but not with age, AL or C/D ratio. Contrastingly, Totan *et al*.^22^ found that average GCIPL was related to age (β, 0.226; P = 0.009) and AL (β, −1.537; P < 0.001), not to SE, rim area or disc area, while Goh *et al*.^23^ determined that average GCIPL was correlated with AL (β, −2.056; P < 0.001) though not with age.

We found, in sum, that the higher the myopia was, the thinner the GCIPL thickness was; also, we uncovered a positive correlation between macular GCIPL thickness and peripapillary RNFL thickness.

The present study has some limitations. First, as all of our study subjects were of Korean ethnicity, we could not examine any possible inter-racial relationships. Second, we included subjects representative of the full range of refractive errors in order to investigate the association between average GCIPL thickness and SE. As such, high myopia (≤ −6.0D) or high hyperopia (≥ + 6.0D) could be a cause of possible bias incurred in obtaining the normative data.

In conclusion, this study is, as far as we know at least, the first to have evaluated normative values of GCIPL thickness in healthy children using SS-OCT. The information obtained can provide an SS-OCT-based pediatric normative database of GCIPL thickness that can serve as a reference for early detection and follow-up of glaucoma and other optic nerve diseases in children.
